# Genotypic and phenotypic heterogeneity among Chinese pediatric genetic white matter disorders

**DOI:** 10.1186/s13052-023-01555-z

**Published:** 2023-11-19

**Authors:** Liling Dong, Li Shang, Caiyan Liu, Chenhui Mao, Xinying Huang, Shanshan Chu, Bin Peng, Liying Cui, Jing Gao

**Affiliations:** Neurology department, State Key Laboratory of Complex Severe and Rare Diseases, Peking Union Medical College Hospital, Chinese Academy of Medical Sciences and Peking Union Medical College, Shuaifuyuan No. 1, Dongcheng district, Beijing, 100005 China

**Keywords:** Pediatric genetic white matter disorder, Genetic spectrum, *ABCD1*

## Abstract

**Background:**

The pediatric genetic white matter disorders are characterized by a broad disease spectrum. Genetic testing is valuable in the diagnosis. However, there are few studies on the clinical and genetic spectrum of Chinese pediatric genetic white matter disorders.

**Methods:**

The participants were enrolled from the cohort of Peking Union Medical College Hospital. They all received history collection, brain MRI and gene sequencing. Their neurologic complaints which were related to white matter disorders occurred before 18. Brain MRI indicated periventricular and/or deep white matter lesions, fazekas grade 2–3.

**Results:**

Among the 13 subjects, there were 11 males and two females. The average age of onset was 10.0 ± 5.5 years old. The potential genetic variants were found in 84.6% (11/13) subjects. The *ABCD1* showed the greatest mutation frequency (30.8%, 4/13). The *EIF2B3* A151fs, *EIF2B4* c.885 + 2T > G, *EIF2B5* R129X and *MPV17* Q142X were novel pathogenic/likely pathogenic variants. 100% (4/4) *ABCD1* carriers were accompanied by visual impairment, whereas 100% (3/3) *EIF2B* carriers developed dysuria. 100% (4/4) *ABCD1* carriers exhibited diffuse white matter hyperintensities mainly in the posterior cortical regions, while the *EIF2B4* and *EIF2B5* carriers were accompanied by cystic degeneration.

**Conclusion:**

There is genotypic and phenotypic heterogeneity among Chinese subjects with pediatric genetic white matter disorders. The knowledge of these clinical and genetic characteristics facilitates an accurate diagnosis of these diseases.

**Supplementary Information:**

The online version contains supplementary material available at 10.1186/s13052-023-01555-z.

## Background

The genetic white matter disorders are a group of heterogeneous diseases that predominantly affect the white matter of central nervous system. The clinical onset usually occurs in childhood. The motor impairment is the most prevalent, followed by cognitive deficit, behavioral abnormality, seizure, etc. [1]. On MRI, there is T2 hyperintensity in the white matter with T1 hypo-, iso- or hyperintensity relative to the gray matter [2].

According to Vanderver’s definition and classification, the genetic white matter disorders involve leukodystrophies and genetic leukoencephalopathies. Leukodystrophies include X-linked adrenoleukodystrophy, metachromatic leukodystrophy and Krabbe leukodystrophy, etc. And genetic leukoencephalopathies include Fabry disease, mitochondrial disorders, etc. [2].

These diseases are heterogeneous and overlapping in phenotype. Some have characteristic enzymatic alterations, such as the elevated very long chain fatty acids (VLCFA) in X-linked adrenoleukodystrophy [3]. Some do not, such as the vanishing white matter diseases [4]. Therefore, genetic testing is valuable in the diagnosis of genetic white matter disorders.

Many studies focused on the clinical and genetic spectrum of these diseases. In a UK pediatric cohort with leukodystrophies and genetic leukoencephalopathies (n = 803), the clinical spectrum involved mucopolysaccharidoses (12.5%, 100), GM1/GM2 gangliosidoses (11.3%, 91), metachromatic leukodystrophy (9.5%, 76), adrenoleukodystrophy (9.2%, 74), Krabbe leukodystrophy (6.8%, 55), etc. [5]. In a Iranian pediatric cohort with leukodystrophies and leukoencephalopathies (n = 152), the most common disease was metachromatic leukodystrophy (12.5%, 19), followed by Canavan disease (7.9%, 12), Tay-Sachs disease (7.2%, 11), adrenoleukodystrophy (5.3%, 8), Pelizaeus–Merzbacher like disease type 1 (5.3%, 8), etc. [6]. The genetic markers involved the *ABCD1*, *EIF2B*, *PLP1*, *ARSA*, *MLC1*, *GALC*, *ASPA* and *GFAP* genes, etc. [5, 6].

However, there are few studies on the clinical and genetic spectrum of Chinese pediatric genetic white matter disorders. This is a retrospective study from the cohort of Peking Union Medical College Hospital (PUMCH). Herein, we will describe the clinical and genetic characteristics of Chinese pediatric patients with genetic white matter disorders.

## Method

### Participants

The participants were enrolled from PUMCH cohort. The inclusion criteria were as following: ① Intact data on clinical history, brain MRI and gene sequencing. The MRI sequences included T1/T2 weighted, fluid-attenuated inversion recovery (FLAIR) and diffusion-weighted images (DWI). ② The neurologic complaints which were related to white matter disorders occurred before 18. The neurologic symptoms or signs could be developmental delay, intellectual disability, speech difficulty, psychiatric disturbance, motor regression, gait ataxia, spasticity, hearing or visual loss, autonomic dysfunction, seizure, etc. ③ Brain MRI indicated periventricular and/or deep white matter lesions, fazekas grade 2–3. The white matter lesions presented with T2/FLAIR hyperintensity with T1 hyper-, iso- or hypointensity.

The subjects were excluded if they were better explained by acquired white matter disorders due to infection, toxicity, ischemia with large or medium vessel occlusion, nutritional deficiency, trauma, neoplasm or systemic autoimmune disease.

As illustrated in Supplement Fig. 1, there were 14 pediatric patients with genetic white matter disorders. One 17-year-old female was further excluded since she had periventricular white matter lesions (fazekas grade 2), but no clinical symptom or sign. Finally, 13 subjects were enrolled.

### Gene sequencing

Three cases received whole exome sequencing. Ten subjects had targeted exome sequencing of 278 genes which were related to dementia and white matter disorders. The next-generation sequencing were performed on the Illumina platform (Illumina Inc., San Diego, CA, USA). The pathogenicity of the variants was interpreted using the standards of American College of Medical Genetics and Genomics (ACMG) [7].

## Result

### Demographics

As shown in Tables 1 and 13 subjects of Chinese ancestry were enrolled, including 11 males and two females. The age of onset ranged between ten months and 17 years, with an average of 10.0 ± 5.5 years old. 12 cases were *APOE*-ε4 non-carriers, and one was ε4ε4 genotype. The elder brother of Case 1 was diagnosed with adrenoleukodystrophy at eight and died at 10. As for the other 12 subjects, no similar clinical manifestations were shown among their first-degree or second-degree family members (Table 2).

**Table 1:** Demographics of 13 participants with pediatric genetic white matter disorders

	Participants (n = 13)
Male/Female n(%)	11 (84.6%) / 2 (15.4%)
Age (years old)	17.0 ± 9.7
Age of onset (years old)	10.0 ± 5.5
Family history (+/-) n(%)	1 (7.7%) / 12 (92.3%)
Motor disorder (+/-) n(%)	9 (69.2%) / 4 (30.8%)
Cognitive impairment (+/-) n(%)	11 (84.6%) / 2 (15.4%)
Behavioral abnormality (+/-) n(%)	2 (15.4%) / 11 (84.6%)
Seizure (+/-) n(%)	2 (15.4%) / 11 (84.6%)
Dysuria (+/-) n(%)	3 (23.1%) / 10 (76.9%)
Visual impairment (+/-) n(%)	4 (30.8%) / 9 (69.2%)
Auditory impairment (+/-) n(%)	2 (15.4%) / 11 (84.6%)
*APOE*-ε4 (+/-) n(%)	1 (7.7%) / 12 (92.3%)
Causative mutation (+/-) n(%)	11 (84.6%) / 2 (15.4%)

**Table 2:** Clinical characteristics of 13 participants with pediatric genetic white matter disorders

Case	Gender	Age/AOO	Clinical symptom	Brain MRI	*APOE*
1	Male	11/10	Progressive visual impairment, speech problem, adrenocortical insufficiency	Bilateral WMH in parieto-occipital region	ε2ε3
2	Male	28/13	Progressive speech problem, memory deficit, visuospatial impairment, ataxia, adrenocortical insufficiency	Bilateral WMH in parieto-occipital region	ε3ε3
3	Male	11/10	Progressive intellectual impairment, visual and auditory impairment, dysarthria, ataxia, spastic paraplegia, adrenocortical insufficiency	Bilateral WMH in parieto-occipital region, degeneration of corticospinal tracts in brainstem	ε2ε2
4	Male	14/12	Progressive intellectual impairment, visual impairment, ataxia, spastic paraplegia	Bilateral WMH in tempo-parieto-occipital region, splenium of corpus callosum	ε3ε3
5	Male	17/16	Intellectual impairment, dysuria after syncope	Bilateral WMH in centrum semiovale, corona radiata	ε3ε3
6	Male	29/7	Progressive ataxia, rapid deterioration of cognitive function, psychosis, spastic tetraplegia, dysuria after trauma	Bilateral diffuse WMH in centrum semiovale, corona radiata, middle cerebellar peduncles, cystic degeneration	ε3ε3
7	Female	22/12	Progressive intellectual impairment, spastic tetraplegia, dysarthria, dysuria, seizure	Bilateral diffuse WMH in centrum semiovale, corona radiata, middle cerebellar peduncles, cystic degeneration, restricted diffusion	ε3ε3
8	Male	2/1.5	Motor regression, spastic tetraplegia, dysarthria, feeding difficulties	Bilateral diffuse WMH in centrum semiovale, corona radiata, splenium of corpus callosum, restricted diffusion, stripe-like pattern	ε3ε3
9	Male	28/17	Progressive cognitive impairment, pyramidal sign	Bilateral diffuse WMH in periventricular area	ε3ε3
10	Male	13/3	Motor development delay, intellectual disability, seizure	Bilateral diffuse WMH in centrum semiovale, corona radiata, corpus callosum, middle cerebellar peduncles	ε3ε3
11	Male	15/12	Progressive intellectual impairment, peripheral neuropathy, pyramidal sign	Diffuse WMH in subcortical regions and cerebellum, restricted diffusion	ε3ε3
12	Male	1.5/0.8	Developmental retardation, optic atrophy	Bilateral diffuse WMH in centrum semiovale, corona radiata	ε3ε3
13	Female	29/16	Auditory impairment, psychosis after delivery, dysplasia of left femur head, deformity of left toe	Bilateral diffuse WMH in centrum semiovale, corona radiata, cerebellum, brainstem	ε4ε4

AOO, age of onset; *APOE*, apolipoprotein e; WMH, white matter hyperintensities

### Mutation interpretation

18 variants were found, including the *ABCD1* (n = 4), *EIF2B3* (n = 2), *EIF2B4* (n = 2), *EIF2B5* (n = 2), *ARSA* (n = 2), *GFAP* (n = 1), *NDUFS1* (n = 2) and *MPV17* variants (n = 3) (Table 3).

**Table 3:** Genetic findings of 11 participants with pediatric genetic white matter disorders

Case	Gene	Mutation	1000 g/ESP6500 /GnomAD	SIFT/Polyphen2 /MutationTaster	Clinvar	ACMG	PMID
1	*ABCD1* (NM_000033)	c.1415_1416del p.Q472fs	-/-/-		Pathogenic	Pathogenic	7,849,718
2		c.520T > G p.Y174D	-/-/-	D/D/A	Pathogenic	Likely pathogenic	7,849,723
3		c.796G > A p.G266R	-/-/-	D/D/A	Pathogenic	Likely pathogenic	9,195,223
4		c.1028G > A p.G343D	-/-/-	D/D/D	Likely pathogenic	VUS	
5	*EIF2B3* (NM_020365.4)	c.130G > A p.E44K	0.0002/-/0.000008	D/D/D	VUS	VUS	34,755,279
		c.450dupA p.A151fs	-/-/-		Likely pathogenic	Likely pathogenic	
6	*EIF2B4* (NM_001034116.1)	c.1337G > A p.R446H	-/-/0.00006	D/D/D		VUS	35,860,328
		c.885 + 2T > G	-/-/-			Pathogenic	
7	*EIF2B5* (NM_003907)	c.C385T p.R129X	-/-/0.000004		Likely pathogenic	Pathogenic	
		c.G633T p.R211S	-/-/-	D/P/D		VUS	
8	*ARSA* (NM_000487.5)	c.448 C > T p.P150S	-/-/-	D/D/D	VUS	VUS	
		c.242G > A p.G81D	-/-/-	D/D/D		VUS	
9	*GFAP* (NM_002055.4)	c.1246 C > T p.R416W	-/0.0005/0.00003	D/D/D	Pathogenic	Likely pathogenic	16,826,512
10	*NDUFS1* (NM_005006.6)	c.266T > A p.V89E	-/-/-	D/P/D		VUS	
		c.1609 A > C p.I537L	-/-/0.00003	T/B/D		VUS	
11	*MPV17* (NM_002437)	c.A263T:p.K88M	-/-/0.00006	D/D/D	Likely pathogenic	VUS	22,964,873
		c.C424T:p.Q142X	-/-/-			Likely pathogenic	
		c.A265T:p.M89L	-/-/0.00005	D/P/D		VUS	

VUS, variants of uncertain significance

According to the ACMG criteria, there were eight pathogenic or likely pathogenic variants. Of them, the *ABCD1* Q472fs, Y174D, G266R and *GFAP* R416W were reported before [8–11]. The *EIF2B3* A151fs, *EIF2B4* c.885 + 2T > G, *EIF2B5* R129X and *MPV17* Q142X were novel. The other ten missense variants were variants of uncertain significance (VUS). Of them, the *EIF2B3* E44K, *EIF2B4* R446H and *MPV17* K88M were reported before [12–14]. The *ABCD1* G343D, *EIF2B5* R211S, *ARSA* P150S, G81D, *NDUFS1* V89E, I537L and *MPV17* M89L were novel.

#### *ABCD1* carriers

**Fig. 1:**
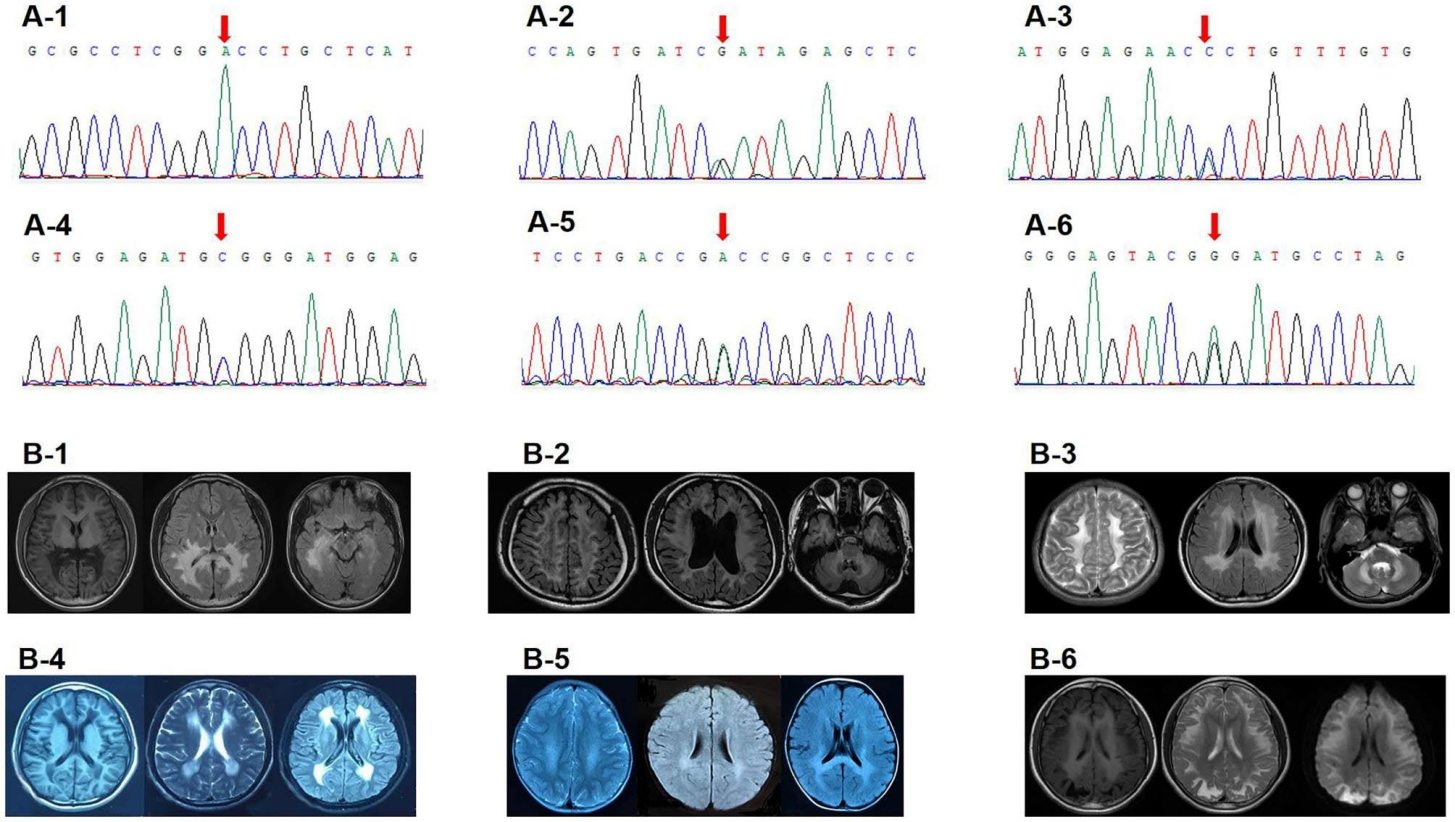
Sanger sequencing and Brain MRI examples. (**A-1**) Sanger sequencing showed *ABCD1* c.1028G > A (p.G343D) in Case 4. (**A-2/3**) Sanger sequencing showed *EIF2B5* c.C385T (p.R129X) and c.G633T (p.R211S) in Case 7. (**A-4**) Sanger sequencing showed *GFAP* c.1246 C > T (p.R416W) in Case 9. (**A-5/6**) Sanger sequencing showed *ARSA* c.242G > A (p.G81D) and c.448 C > T (p.P150S) in Case 8. (**B-1**) Brain MRI showed bilateral WMH in tempro-parieto-occipital region and splenium of corpus callosum in Case 4. (**B-2**) Brain MRI showed bilateral diffuse WMH in the centrum semiovale, corona radiata, middle cerebellar peduncles with cystic degeneration in Case 6. (**B-3**) Brain MRI showed bilateral diffuse WMH in the centrum semiovale, corona radiata, corpus callosum, middle cerebellar peduncles in Case 10. (**B-4**) Brain MRI showed bilateral diffuse WMH in the periventricular area in Case 9. (**B-5**) Brain MRI showed bilateral T2/DWI hyperintensities in the centrum semiovale, corona radiata, splenium of corpus callosum with stripe-like pattern in Case 8. (**B-6**) Brain MRI showed diffuse T2/DWI hyperintensities in the subcortical regions, and T1 hyperintensities in the periventricular areas in Case 11

Four male subjects harbored the *ABCD1* variants. They all had elevated VLCFA. Three of them were accompanied by adrenocortical insufficiency. Brain MRI mainly showed bilateral posterior white matter hyperintensities (WMH) (Fig. 1).

The Q472fs carrier presented with visual and linguistic impairment at 10. He could speak fluently, but had difficulty in oral comprehension. The Y174D carrier started with difficulty in oral comprehension at 13. Gradually he presented with ataxia, memory deficit and visuospatial impairment. The G266R carrier showed intellectual impairment at 10. Gradually he exhibited ataxia, spastic paraplegia, dysarthria, visual and auditory impairment. He was bedridden at 13. The G343D carrier started with intellectual impairment at 12. Afterwards, he developed ataxia, spastic paraplegia and visual impairment. His serum cortisol and ACTH were normal.

#### *EIF2B* carriers

Three subjects harbored the *EIF2B* variants. They all had normal serum VLCFA, homocysteine (HCY), folic acid, vitamin B12 and organic acids. The brain MRI mainly indicated bilateral WMH in the centrum semiovale and corona radiata (Fig. 1). Two subjects had coexisting cystic degeneration.

The *EIF2B3* E44K (paternal origin) and A151fs (maternal origin) were found in a 17-year-old male. He presented with intellectual impairment and dysuria after a syncope at 16. The *EIF2B4* R446H (maternal origin) and c.885 + 2T > G (de novo) were in a 29-year-old male. He presented with progressive ataxia since seven. He could run and climb, but fell frequently. After an accidental trauma at 28, he showed unsteadiness while standing. Gradually, he developed memory deficit, disorientation, dyscalculia, delusion, spastic tetraplegia and dysuria. The *EIF2B5* R211S (paternal origin) and R129X (de novo) were detected in a 22-year-old female. She started with seizure attack at 12. Gradually, she showed poor academic performance at school, accompanied by spastic tetraplegia, dysarthria and dysuria. At 20, she could not vocalize, eat or move.

#### *ARSA*, *GFAP*, *NDUFS1* and *MPV17* carriers

Four subjects carried the variants in the *ARSA*, *GFAP*, *NDUFS1* and *MPV17*. They had normal serum VLCFA, HCY, folic acid, vitamin B12 and organic acids.

The *ARSA* P150S (paternal origin) and G81D (maternal origin) were found in a two-year-old boy. At the age of one, he was able to walk and call mom, dad. Four months later, he showed gait instability and dysarthria. Gradually he could not speak, eat or move. Physical examination indicated spastic tetraplegia. Brain MRI showed bilateral diffuse WMH in the centrum semiovale, corona radiata, splenium of corpus callosum with stripe-like pattern (Fig. 1). Serum enzyme test showed decreased Arylsulfatase A level (3nmol/17 h/mgPr).

The de novo *GFAP* R416W was in a 28-year-old male. He showed cognitive decline since 17. Physical examination indicated bilateral Babinski sign with normal muscle force. Brain MRI revealed bilateral diffuse WMH in the periventricular area (Fig. 1).

The *NDUFS1* V89E and I537L were in a 13-year-old boy. He was able to walk at three. He showed poor intelligence and seizure attack in early childhood. Brain MRI indicated bilateral diffuse WMH in the centrum semiovale, corona radiata, corpus callosum and middle cerebellar peduncles (Fig. 1).

The *MPV17* Q142X, M89L and K88M were in a 15-year-old boy. He showed poor academic performance at 12. Two years later, he developed limb weakness and gait instability. Physical examination exhibited bilateral Babinski sign, decreased muscle force and tendon reflex in the extremities, as well as reduced pin-prick sensation in the distal extremities. Electromyography revealed peripheral neuropathy. Brain MRI demonstrated T2/DWI hyperintensities in the subcortical regions, cerebellum, and T1 hyperintensities in the periventricular areas (Fig. 1).

## Discussion

This is a group of subjects with pediatric genetic white matter disorders. The potential genetic variants are found in 84.6% (11/13) subjects. The *ABCD1* has the greatest mutation frequency (30.8%, 4/13), followed by the *EIF2B* (23.1%, 3/13) and mitochondrial genes (15.4%, 2/13), which suggest the diagnosis of adrenoleukodystrophy, vanishing white matter disease and mitochondrial disease, respectively. These three diseases are also the main components of pediatric leukoencephalopathies in other countries. In a Finnish pediatric cohort with genetic white matter disorders, the most common diseases are mitochondrial (18.8%, 15/80) and adrenoleukodystrophy (7.5%, 6/80) [15]. In English and Iranian pediatric cohorts with leukoencephalopathies, the prevalence of vanishing white matter disease are 18.8% (17/903) and 2.6% (4/152), respectively [5, 6].

11 novel variants are found in this report. Of them, the *EIF2B3* A151fs, *EIF2B4* c.885 + 2T > G, *EIF2B5* R129X, *MPV17* Q142X are frameshift, splicing or stopgain variants, They are rare or absent in the 1000genome, ESP6500, GnomAD databases. The *EIF2B4* c.885 + 2T > G and *EIF2B5* R129X are de novo based on the pedigree analysis. The *EIF2B3* A151fs and *EIF2B5* R129X are determined as likely pathogenic in the Clinvar database (www.clinvar.com). Taken together, these variants are pathogenic/likely pathogenic according to the ACMG criteria.

Seven novel VUS are detected, including the *ABCD1* G343D, *EIF2B5* R211S, *ARSA* P150S and G81D, *NDUFS1* V89E/I537L and *MPV17* M89L. They are rare or absent in the 1000genome, ESP6500, GnomAD databases. They are deleterious from SIFT, Polyphen2 and Mutationtaster predictions. The *ARSA* P150S is novel, while the *ARSA* P150L is reported before [16]. The latter is supposed to be pathogenic/likely pathogenic in the clinvar database (www.clinvar.com). The subjects harboring the *ABCD1* G343D and the *ARSA* P150S/G81D demonstrate increased VLCFA and decreased Arylsulfatase A levels, respectively. Taken together, these variants are VUS according to the ACMG criteria.

There are some common phenotypic features among the 13 subjects. For instance, 76.9% (10/13) cases are characterized by insidious onset and gradual progression. The most prevalent symptoms are cognitive impairment (84.6%, 11/13) and motor disorder (69.2%, 9/13). 100% (13/13) subjects have diffuse WMH in the supratentorial subcortical or periventricular regions. These are almost consistent with previous research [1, 2].

Moreover, the subjects with different gene mutations have some characteristic phenotypes. For instance, none of the three *EIF2B* variant carriers exhibit a typical pattern of insidious onset and gradual progression. The *EIF2B5* carrier presents with an acute onset of seizure attack. The *EIF2B3* and *EIF2B4* carriers experience a rapid progression of neurological regression following syncope and trauma. Previous reports reveal that patients with vanishing white matter diseases can worsen rapidly under stress [4]. The mechanism is unknown.

In terms of clinical symptom, 100% (4/4) *ABCD1* carriers are accompanied by visual impairment. This might be related to their occipital involvement. The *MPV17* carrier has peripheral neuropathy. El-Hattab pointed out that peripheral neuropathy occurred in 18.7% (17/91) *MPV17* carriers [17]. In addition, we find 100% (3/3) *EIF2B* carriers develop dysuria. Kar found that the mRNAs encoding the EIF2B2 were present in the axons of rat sympathetic neurons [18]. These lead to the speculation that the *EIF2B* genes might exert effects on autonomic nerves.

On brain MRI, 100% (4/4) *ABCD1* carriers exhibit diffuse WMH mainly in the posterior cortical regions. The *EIF2B4* and *EIF2B5* carriers are accompanied by cystic degeneration. The *ARSA* carrier shows stripe-like pattern, indicating remaining tissue strands within the WMH. The T2 hyperintensity of the *MPV17* carrier is primarily limited in the subcortical regions. These are similar to previous findings [3, 4, 19, 20]. However, the mechanism remains unclear.

There are two subjects carrying the variants related to mitochondrial diseases. The *NDUFS1* carrier presents with motor development delay, intellectual disability, seizure with diffuse cerebral and cerebellar WMH. According to Björkman’s finding, these clinical and imaging features are common in the *NDUFS1* carriers except for the cerebellar involvement [21]. The association between the *NDUFS1* gene and cerebellum could be further investigated. Fortunately, the *MPV17* carrier has a late onset without hepatic impairment. El-Hattab summarized the clinical characteristics of 100 *MPV17* mutation carriers. He found that almost 96% individuals had an poor prognosis during infancy or early childhood due to hepatic failure. However, 4% subjects with later onset during late childhood or adulthood had no or minimal liver injury [17].

## Conclusions

There is genotypic and phenotypic heterogeneity among Chinese pediatric genetic white matter disorders. The *ABCD1*, *EIF2B* and mitochondrial genes have high mutation frequencies. The subjects with different gene mutations exhibit characteristic manifestations, which have suggestive implications for the underlying genetic basis. The knowledge of these clinical and genetic characteristics facilitates an accurate diagnosis of these diseases. The functional verification of the novel variants should be performed in the following studies.

### Electronic supplementary material

Below is the link to the electronic supplementary material.

Supplementary Material 1

Supplementary Material 2

## Data Availability

The original contributions are included in the article, further dataset are available from the corresponding author on reasonable request.

## Abbreviations

VLCFA: Very long chain fatty acids
PUMCH: Peking Union Medical College Hospital
ACMG: American College of Medical Genetics and Genomics
VUS: Variants of uncertain significance
HCY: Homocysteine
WMH: White matter hyperintensities
