# Supplementary figures and images for: Genotypic and phenotypic heterogeneity among Chinese pediatric genetic white matter disorders
Source: Ital J Pediatr. 2023 Nov 19;49:155. doi: 10.1186/s13052-023-01555-z (PMC10658925; doi:10.1186/s13052-023-01555-z)

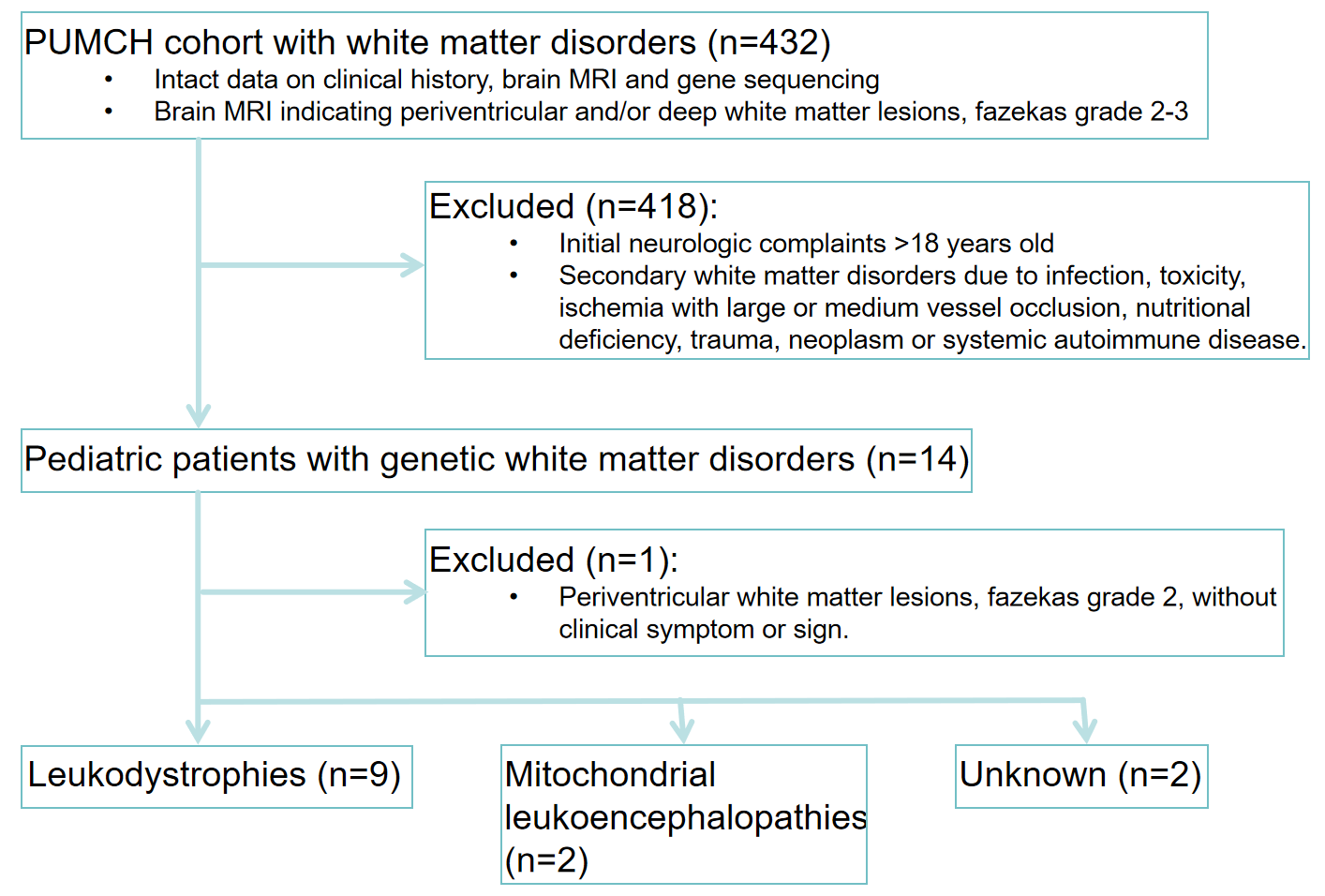


Supplement Figure 1: Flow chart of the study

Supplement: Supplementary file 1 — Supplementary Material 1 [file 13052_2023_1555_MOESM1_ESM.doc]
