# Supplementary material for: Genotypic and phenotypic heterogeneity among Chinese pediatric genetic white matter disorders
Source: Ital J Pediatr. 2023 Nov 19;49:155. doi: 10.1186/s13052-023-01555-z (PMC10658925; doi:10.1186/s13052-023-01555-z)
Supplement: Supplementary file 2 — Supplementary Material 2 [file 13052_2023_1555_MOESM2_ESM.doc]

Reviewer #1: goood

Reviewer #2: I congratulate you on your work, the topic you chose, and the writing of the article.

I suggest better specifying the clinical and imaging enrollment criteria, paying attention to the MRI patterns examined.

**Answer:**

- We have revised the clinical and imaging enrollment criteria (METHOD/Participants, in RED). And we added the flow chart in the Supplement.

Also, I think it is useful to better specify the age range examined by dwelling on the greater definition of "preadults."

**Answer:**

- We have changed the word “preadult” into “pediatric”. Thanks for your guidance. And the age range was described in “METHOD/Participants/②” (in RED).

Reviewer #3: We appreciate the time and effort it takes to submit your work

The manuscript is interesting and has good readability

However, the criterion for enrolling the 13 patients should be clarified and the clinical impact of the results discussed in more detail

**Answer:**

- We have revised the clinical and imaging enrollment criteria (METHOD/Participants, in RED). And we added the flow chart in the Supplement.
- The clinical impact of the results were further discussed in DISCUSSION section. We discussed the mutation frequency (1^st^ paragraph), novel pathogenic/likely pathogenic variants (2^nd^ paragraph), novel variants of uncertain significance (3^rd^ paragraph), common phenotypic features (4^th^ paragraph), characteristic phenotypes in onset and disease course (5^th^ paragraph), characteristic clinical symptom (6^th^ paragraph), characteristic brain MRI (7^th^ paragraph), and the rare manifestations of two mitochondrial cases (8^th^ paragraph, in RED).
